# Beyond wilderness: towards an anthropology of infrastructure and the built environment in the Russian North[Fn FN0001]

**DOI:** 10.1080/2154896X.2017.1334427

**Published:** 2017-06-09

**Authors:** Peter Schweitzer, Olga Povoroznyuk, Sigrid Schiesser

**Affiliations:** ^a^ Department of Social and Cultural Anthropology, University of Vienna, Vienna, Austria

**Keywords:** Arctic, Soviet Union, Russian Federation, infrastructure, built environment, transportation, Baikal-Amur Mainline, Northern Sea Route

## Abstract

Public and academic discourses about the Polar regions typically focus on the so-called natural environment. While, these discourses and inquiries continue to be relevant, the current article asks the question how to conceptualize the on-going industrial and infrastructural build-up of the Arctic. Acknowledging that the “built environment” is not an invention of modernity, the article nevertheless focuses on large-scale infrastructural projects of the twentieth century, which marks a watershed of industrial and infrastructural development in the north. Given that the Soviet Union was at the vanguard of these developments, the focus will be on Soviet and Russian large-scale projects. We will be discussing two cases of transportation infrastructure, one of them based on an on-going research project being conducted by the authors along the Baikal–Amur Mainline (BAM) and the other focused on the so-called Northern Sea Route, the marine passage with a long history that has recently been regaining public and academic attention. The concluding section will argue for increased attention to the interactions between humans and the built environment, serving as a kind of programmatic call for more anthropological attention to infrastructure in the Russian north and other polar regions.

## Introduction

While public imaginations of the Arctic and Antarctic continue to be fed by notions of unspoiled wilderness, empty expanses and drowning polar bears, the polar regions of the world have long been sites of industrial resource extraction and other activities requiring large-scale infrastructures. While anthropologists and other social scientists have long argued that “wilderness” is a western concept alien to the indigenous inhabitants of the polar regions,^1^ the focus of research has nevertheless remained on human–environmental relations, including perturbations of the natural environment through climate change and other factors. Also, it should be mentioned that “wilderness” is an even more important concept in Antarctic regions^2^ than in Arctic ones, the latter being the focus of this article. At the same time, “wilderness” is used here primarily as a stand-in for “natural environments,” without focusing on the semantic differences these notions might imply.

More recently, the topic of the relations between extractive industries and local residents has started to attract the attention of anthropologists and other social scientists. While there have been many important works on this topic outside the polar regions,^3^ recently the Arctic also has become represented in these investigations.^4^ Still, while large-scale infrastructures are a necessity for these extractive industries – no matter whether they are based on oil and gas, mining, or other resources – the studies in question are less directed at the human-infrastructure nexus than at the social relations between companies and local communities or between employers and employees at these sites of industry.

This article is intended as a programmatic call for more anthropological attention to infrastructure and other elements of the built environment in the polar regions. It has the twofold task of illustrating historical and present-day infrastructure projects in the Arctic and of demonstrating a number of disciplinary approaches and methodological tools in infrastructure studies that could be applied in the focus regions. Before doing so, we will provide a brief overview over anthropological and interdisciplinary treatments of infrastructure and the built environment. Drawing on case studies from the field of transportation and industrial infrastructures in the Arctic we will focus on Russia as, the most urbanised and, by far, the most industrialised part of this region.

### Infrastructure and the built environment in anthropology

The notion of the “built environment” has both the advantage and disadvantage of being a broad and imprecise label, which refers to elements in the environment not considered “natural”. While any strict dichotomy between “natural” and “built” must falter in the light of their entanglements, we use the term in order to situate “infrastructure” within the broader sphere of human–environmental interactions. As the notion of infrastructure was our starting point in thinking through the issues at hand, we will start with a brief discussion of the latter term and then contextualise it as part of the “built environment”.

A recent review of anthropological literature on infrastructure^5^ demonstrates two findings relevant for our purposes: the anthropological study of infrastructure is a fairly recent phenomenon and it has happened largely outside of the polar regions. While Larkin makes references back to Marx, Heidegger, Benjamin and Mumford, the overwhelming majority of his references date from the last 20 years. Although one late twentieth century article carries “ethnography of infrastructure”^6^ in its title, it would be misleading to ascribe it to anthropology in a narrow disciplinary sense. The late Susan Leigh Star was a sociologist, science and technology studies and information science scholar.

While it can be argued that “infrastructure” had been ignored by anthropology for a long time, there has been a noticeable increase in the number of anthropological contributions to it more recently.^7^ One of the areas that are of most interest to us is the exploration of the relationship between humans and infrastructure. The examples range from addressing the legacy of Soviet modernity in recent studies of post-Soviet realities,^8^ while older social science and humanities studies of transportation infrastructures elsewhere document their impact on the notions of speed, distance and space.^9^ Although the study of infrastructure may be “boring” and “unexciting”,^10^ it is of critical importance for our interests, given that “infrastructures are matter that enable the movement of other matter”.^11^ The notion of “infrastructural violence”^12^ is particularly relevant for us given its attention to mechanisms of inclusion and exclusion, that is relations of inequality and marginalisation through infrastructure, which could be seen as description of relations with indigenous groups in the circumpolar North.

Another useful concept is that of the “technosocial”, which was introduced in order to analyse how technology and humans co-operate within specific social settings.^13^ The term seems particularly useful in addressing the interrelationship and mutual constitution of technology, infrastructure and humans. Michael Fisch, who studied the interconnectedness of humans and technologies through the example of Tokyo’s commuter trains, suggests that social and technological situations must be understood coevally.^14^ It should be noted, however, that one of the potential pitfalls of the increasing interest in the notion of “infrastructure” and related concepts is the danger to enlarge the meaning of infrastructure to the point that it can denote any aspect of technologically-driven alterations of the environment.

A recent article exploring the history of the term infrastructure reminds us that the word entered the English-language vocabulary in 1927, or possibly a few decades earlier, from French in order to denote construction and organisational work at a railroad project that was conducted beneath unlaid tracks and prior to building structures above the tracks.^15^ Carse argues that this engineering term owes a lot of its twentieth century success story in the social sciences to the impact of French structuralism and Marxism, both of which assumed that deep structures beyond the observable are what really matters and constitute the task of the researcher.^16^ While current engagements with the notion of infrastructure seem to be informed by the acknowledgement that it is a “mix of materials, practices, and meanings”, the danger is “to neglect the *infra* in infrastructure”.^17^

In short, infrastructure loses its analytical advantage if everything becomes infrastructure. Thus, we feel the need to position (physical)^18^ infrastructure within the broad realm of the “built environment”.^19^ Unlike the recent popularity of infrastructure, the notion of the built environment has not seen a comparable surge in use in recent years. One of the reasons for that might be the influential writings on the “dwelling perspective” by Tim Ingold.^20^ Especially, his influential essay “Building, dwelling, living: how animals and people make themselves at home in the world”^21^ can be seen as a brilliant argument against the distinction of natural from built environments and, thus, as a rejection of the latter. Ingold’s goal in this essay and elsewhere is to substitute what he calls the “building perspective” with a “dwelling perspective”. His aim in this endeavour, however, is not so much the process of building itself but the supposed distinctive characteristics between human and non-human organisms, including human intentionality (in building). Human uniqueness is not central to our argument; on the contrary, we see no reason to limit the physical alteration of the natural environment to human actors. In order to define “built environment”, we adopt Rapoport’s notion of the “hardware”^22^ of “inanimate components of the environment”.^23^

Historically, the notion of the “built environment” has been tied to anthropological interests in architecture, especially in urban settings.^24^ We believe that the materiality of architecture and the built environment as well as their becoming, consumption and entanglement with politics, historical contexts and globalisation require more anthropological attention. We need to know better how and under which circumstances humans shape their own material environments and how these again make individuals and a society. Caroline Humphrey stated back in 1988, that architecture and the built environment as subjects per se have been rather understudied.^25^ Since then, a number of influential contributions have been published.^26^

A very good overview of anthropological studies on architecture has been provided recently by Victor Buchli.^27^ Architecture has been analysed through the prisms of symbolism, cosmology and spirituality.^28^ That the anthropologist’s engagement with the process of “making” architecture can be the key for understanding the built world around us was recently proposed by Tim Ingold.^29^ With their examples from built structures stemming from the Soviet era, both Humphrey and Buchli revealed how ideologies can be manifested in buildings and reciprocally shape people’s perception of the world.^30^ Amos Rapoport analysed the “physical expression of spatial organization” of the built environment as a significant dimension of a society’s organisation of meaning.^31^ Maudlin and Vellinga highlight the aspect of the appropriation and consumption of buildings exemplified by several case studies.^32^

While references to the “built” in general anthropology reach back into the nineteenth century,^33^ the arctic and polar regions have been typically left out of it. This can be paralleled with the virtual absence, or at least very late arrival, of urban studies or urban anthropology in the Arctic.^34^ The fact that relatively few areas of the North are urban effectively led to a situation in which the existing urban spaces in the Arctic have been rarely investigated in the scholarly literature.^35^ The same reasoning seems to have been applied to built environments in general in polar regions. In both cases (cities and the built environment), it is also important to point out that the Russian part of the Arctic is much more urbanised (and is home to many more people, buildings and infrastructural objects) than the North American Arctic.^36^ Before turning more or less exclusively to Russian cases, we will briefly introduce some recent studies on polar infrastructure elsewhere.

### Infrastructure in polar regions: a few recent examples

Situating our interest in infrastructure within the broader definition of the built environment as physical alteration of the natural environment, it follows that polar infrastructure studies cannot be seen as limited to the recent colonial past. Human alterations of the environment are as old as human habitation of the polar regions, and it can be argued that environmental alterations are not limited to human actors. A comprehensive account of indigenous infrastructural and building practices is a desideratum that goes far beyond what this article can achieve. The same is true for a treatment of environmental alterations by non-human actors, even if they were limited to plants and animals. Instead, we will focus on large-scale infrastructural and building projects, which privileges the twentieth and twenty-first centuries.

Notwithstanding our focus on the recent past and present, we want to mention at least one example that reaches further back, namely the International Polar Year 2007/09 project “History of Large Scale Resource Exploitation in Polar Areas” (LASHIPA), which had been directed by the physical geographer and archaeologist Louwrens Hacquebord.^37^ Hacquebord documents that resource extraction in the Atlantic Arctic by external players reaches back into the seventeenth century, when English, Danish and Dutch whaling companies started to compete for contemporary Svalbard and neighbouring islands.^38^ These early ventures were followed by coal mining operations on Spitsbergen in the nineteenth and twentieth centuries.^39^ At the same time, Russian hunting expeditions to Spitsbergen/Svalbard – carried out by private and state enterprises – from the early eighteenth century onwards further contributed to bringing the Arctic into the realm of capitalist resource exploitation.^40^

LASHIPA served as a timely reminder of the usefulness of historical archaeology and historiography in the study of polar infrastructures. The project also contained a number of contributions by Science and Technology Studies (STS) scholars.^41^ Given the ubiquity of science and technology in modern infrastructure projects, STS approaches have become an important dimension for understanding infrastructural plans and practices in polar regions. The pages of this journal are as much witness of that,^42^ as are a number of recent book publications.^43^

The dissolution of the Soviet Union marked the beginning of a new period in the history of the Arctic by overcoming the divisions of the Cold War. New environmental and political concerns joined growing commercial interest in the Arctic’s raw materials to form the basis for new international alliances in the early post-Soviet period. The emergence of the “common” Arctic launched the modernisation and new construction of industrial and transportation infrastructures serving the needs of resource extraction, global trade, search and rescue operations and, to a lesser extent, tourism and local passenger transport. At the same time, a climate for joint international research in the polar regions emerged. It was only in the 1990s that the circumpolar North emerged as a social science domain.^44^ Historians of science use the term “New Arctic” to refer to the current period in circumpolar history set in motion by an unparalleled confluence of political, environmental and economic factors and addressing a global research agenda of climate change, resource development and indigenous issues.^45^

An important aspect of polar infrastructure are telecommunications installations and practices that provide connectivity under the remote conditions of the Arctic and Antarctic. Ruiz provided a recent example focusing on Nunavut in the Canadian Arctic, in which fluidity and friction emerged as key terms.^46^ At the same time, his anthropological field notes at a distance demonstrate the methodological limitations of polar infrastructure studies.^47^

The prominence of the Antarctic as a new political frontier, a social microcosm and a research laboratory has recently been growing. Thus, we want to mention Salazar’s forthcoming article on “Polar Infrastructures” here, as it focuses on the Antarctic.^48^ He presents three digital ethnographic research strategies, situated on the Antarctic Peninsula, where a network of research stations, semi-permanent settlements and other infrastructure, have become instrumental in the establishment of new forms of colonisation and sociality implicated in the making of experiences of community, solidarity and belonging in extreme polar environments. After this brief excursion into the southern hemisphere, we will now focus on our main topic, infrastructure and the built environment within the context of “mastering” the Russian North.

## Soviet industrialisation, modernisation and infrastructure buildup

The Soviet Union, or the Soviet North, is a perfect example of the transformations of the built environment due to an accelerated modernisation agenda. James Scott’s notion of “high modernism”^49^ seems to be a perfect fit for the social engineering and development policies systematically implemented in remote Northern areas by the Soviet state. According to Scott, this is an ideology based on the belief in scientific and technical progress associated with industrialisation in Western Europe and in North America in the period between the 1880s and the First World War. Continued linear progress, the development of scientific and technical knowledge, the expansion of production, the rational design of social order, the growing satisfaction of human needs and an increasing control over nature constitute the core values of modernisation. At the same time, *high modernism* is a quintessential application of the benefits of technical and scientific progress in every field of human activity. In authoritarian states, like the Soviet Union, such application becomes possible due to three main factors: “administrative ordering of nature and society” (rational engineering of all aspects of social life), unrestrained use of the state power for implementing these projects and a weak civil society lacking the capacity to resist those.^50^ As Scott concludes, modernisation schemes have typically taken their most destructive human and natural toll in the authoritarian states of the former Socialist block and the revolutionary Third World, where religions were replaced by a similar faith in the idea of progress vouched by scientists, engineers and planners. In these countries, the intentions to improve the human condition underlying modernisation schemes have failed because their progenitors regarded themselves smarter than their subjects and planned for abstract standardised citizens with no gender, values, opinions or needs.^51^

The Soviet programme of “mastering the North” (*osvoenie Severa*) has become the most vivid expression of the state modernisation plans and infrastructure building in the country’s most remote corners. The definitions of “the North” varied depending on geography (European and Asian North), accessibility by transport (Near and Far North) and population composition and density (Extreme^52^ North and “territories equaled to it”, including areas occupied by indigenous minorities), etc.^53^ The first and foremost purposes of Soviet “mastering” programs have been state control over and economic development of the northern territories. The latter has been focused on the extraction of rich, but hardly accessible, natural resources – from fish, game and forest, to gems and metals, to oil and gas. Whereas harvesting renewable resources has been a long tradition, in the Soviet Union geological prospecting started with large-scale expeditions in the 1920s and 1930s, continued with the exploration of rich oil and gas fields in West Siberia in the 1960s and 1970s and reached well into the period of political and economic changes of the late 1980s and the 1990s. Propaganda campaigns, labour resource mobilisation and, finally, infrastructure build up were the key components of large-scale development projects in the Soviet “mastering the North” programme.

### The ideology of “Mastering the North”

Propaganda discourses underlying the idea of “mastering of the North” were centred around widespread modernist concepts of human dominance over nature. The strict division between “social” and “natural” orders goes back to the Enlightenment construction of Nature as an external and primordial environment to be physically mastered by the growth of urban environments.^54^ From this perspective, the Arctic was constructed as an important frontier and wilderness territory where the natural environment is immensely alien and hostile to humans. Soviet interpretations of the Arctic myth were expressed in such notions “the war against the environment”, “the struggle with the elements”, “the conquest of nature” and others, which were especially popular in the early days of Soviet industrialisation of the North. The dominating discourses of the northern nature have changed with the second wave of resource exploration in the 1960s. The discourse of “the senseless emptiness” implied that only activities of the “civilized” man can endow a certain locality with the “sense” and turn a space into a territory. Other popular discourses of the nature, such as “the treasure-house” or “the warden of treasures”, obviously aimed at attracting people to the North.^55^

Not only in press and media, but also in science and fiction, “nature” was looked upon through the prism of the Soviet polity and economy. Attitudes to industry and resource development, agriculture, ecosystems, biodiversity, urban planning and so on were shaped largely by political and economic concerns.^56^ Similarly to other industrialised countries, the Soviet state looked at the natural environment from the economic perspective oriented at resource extraction.

The idea of dominance over nature was connected to the concept of the “creation of a new Soviet people” imagined as completely separate from the sphere of nature. While the early revolutionary rhetoric presented the nature as an enemy to battle against, in the 1960s, this discourse was standardised to become more pathetic. Soviet people then were depicted as winners and heroes in this struggle against the natural world.^57^ Their heroic qualities were particularly celebrated in the “harsh” and “hostile” conditions of the North.

Soviet “mastering” programs were implemented by a variety of state organisations and institutional actors, which shaped environmental policies and behaviours serving the purposes of a socialist society. Large-scale industrial projects involved big bureaucracies and state organisations. Multiple research institutions and ministries were created to study, transform and manage “nature” in order to force the pace of economic production. Soviet environmental policies were embedded in the broader political, cultural and economic contexts reflecting changing attitudes towards the natural and built environment and resource use.^58^ Soviet infrastructure projects, which were often publicly referred to as “projects of the century”, from roads to plants, to dams, “embraced large-scale technologies with an energy that belied its economic backwardness”. Soviet leaders saw themselves as tools “to convert the largely agrarian, peasant society into a well-oiled machine of workers”^59^ and to efficiently exploit natural resources. Lenin’s electrification, Stalin’s canals, plants and railroads, Khrushchev’s atomic energy, Brezhnev’s river diversion and hydropower stations, etc. expressed constructivist visions of the communist future characteristic for a certain time of the Soviet history.

### Population and labour resources

The population density in the North was generally low in the pre-industrial period, with the majority of residents settling along rivers, post roads and emerging railroads. Therefore, northern territories with a more developed transportation network attracted more newcomers. From this perspective, the famous Transsiberian Railroad (Transsib) played a remarkable role in the continued Russian colonisation of Siberia by expanding the empire’s agriculture, trade and industry along the railroad.^60^ Areas south of the railroad were demographically attractive agricultural zones due to their favourable climatic conditions. While the North had a promising resource potential, it remained primarily occupied by nomadic indigenous groups and had much lower population densities to report.

Since the beginning of Soviet rule, human resources have been a driving force for exploration, industrialisation and infrastructure development in the Russian Arctic. Building socialism implied not only an accumulation of wealth, but also of people. Numerically small and widely dispersed indigenous ethnic groups have been populating the vast territories of the North and Siberia for centuries. The Soviet modernisation project towards indigenous peoples of the North, drew on “civilizing” and enlightenment missions. Thus, it included a series of health, education, culture and economic reforms resulting in drastic and irreversible changes of their migration and settlement patterns and lifestyles. For example, there was a campaign for “cultural construction”, which introduced formal education, written languages, as well as the Soviet mass culture and ideology to indigenous ethnic cultures.^61^ Collectivisation had devastating impacts on traditional economies, nomadic life-styles and settlement patterns. In the 1930s, a large-scale experiment of closing down “unprofitable” communities and their relocation to newly founded settlements severely affected the social and spatial fabric of indigenous communities.^62^

Subsequent agricultural reforms of the 1950s and 1960s changed the perceptions and practices of traditional subsistence activities. For example, with the introduction of the shift method, reindeer herding, the main economic activity for many indigenous groups, has turned from “a way of life” to “a mode of production” resulting in increased sedentarisation and state control over nomadic groups.^63^ Thus, the Sovietisation of the North was a total process of modernisation and industrialisation, executed through different institutions and relying on a technocratic ideology and reorganisation of indigenous subsistence space. At the same time, the construction of infrastructure in the Arctic, respectively the Subarctic, rarely involved indigenous people. New cities, roads, seaports, dams, plants and other industrial objects were typically built by a labour force recruited from other parts of the country.

Labour recruitment and management strategies at the large-scale construction projects evolved with time from forced labour, commonly used during Stalin’s rule, to socialist propaganda in later periods. The 1930s saw a dramatic escalation, of the system of Gulag penal labour, both in size and scope. Stalinism seemed to bring together the conception and experience of what then was propagated as “progressive modernity”, the deployment of a planned economy with large-scale producers and the formation of a government dedicated to the national commonwealth. The American historian Stephen Kotkin describes this era as the time of “intoxication with the power of heavy industry” and “a rallying point for a technologically perfected future”.^64^

Another form of accumulation of labour resources at large-scale construction, which prevailed in later periods of the Soviet rule were so-called mobilisation campaigns. They included ideological, political and, later, material stimuli, which were conducted mostly by the Communist party organisations (such as the *Komsomol*, the Communist youth association of the Soviet Union) and trade unions. Due to their strong ideological, political and institutional underpinning, infrastructure projects themselves were often referred to as “communist construction sites”.^65^ Parallel to these short-term recruitment campaigns, settlement in the North and allocation of labour force for longer term industrial projects were carried out by the state programme of the distribution of specialists. The state guaranteed quotas in key areas in secondary and higher education institutions under the condition that a young specialist will work in a predefined region upon graduation. In the 1950s and 1960s, this system worked well in attracting geologists, engineers, medical doctors, teachers and other key professionals to the areas of planned industrial exploitation. “Northern salaries” or “add-ons” and a number of benefits, including state housing, as well as travel subsidies, offered to the permanent residents of the North have served as tools for retaining the population in the northern areas since the late Soviet period.

Some development projects of the early Soviet industrialisation under Stalin’s regime, such as the construction of the first sections of the Baikal-Amur Mainline (BAM)^66^ and, especially, the White Sea Channel (Belomorkanal) had a notorious reputation for the wide use of forced labour and demonstrated an obscene violation of human rights.^67^ Later projects combined a number of recruitment methods, involving local labour force, as well as attracting migrants from all across the USSR. Yet, all these large-scale engineering projects, regardless of their geography, were the embodiment of Soviet modernisation policies.

Although the case of *Magnitostroi,* a Soviet development project that included the construction of a city with a metallurgic plant doesn’t fit into the “polar” or “northern” geographical scope of this paper, it tells us an illuminating story of other socialist construction sites. Magnitogorsk was the first of the socialist cities (*sotsgoroda* or “blue cities”, using Engel’s terminology^68^) built for resource exploitation and regional development in the 1950–1980s. These cities (Vorkuta, Neryungri, Noril’sk are among the northernmost in this row), share similar urban design and the delineation between public and private spaces embedded in the Soviet ideology. The city construction commenced in 1929, despite the project’s high energy costs and a lack of labour resources.

The plan to build the world’s largest steel plant, near one of the country’s richest and most accessible iron deposits located in West Siberia, epitomised Bolsheviks’ commitment to massive social transformation, mastering the country’s expanse and overcoming Russia’s historical “backwardness”. The city, no less than the plant, played a crucial role in, what Kotkin called, an “internal territorial colonization”.^69^ It included a demographic transformation and the expansion of the country’s industrial and military capacity by creating new urban inhabitants who would operate machines, produce steel and administer the factories.

*Magnitostroy* demonstrated the co-existence of volunteer and forced labour, of heroic self-sacrifice and violent coercion – an “unstable dichotomy that could occur only in a society undergoing a social revolution from below and being simultaneously enslaved from above”.^70^ The labour resources were “mobilized” or sent to the construction site either by a party, government or a trade union order. The recruitment campaign and propaganda in the local and national press targeted the graduates of higher education institutions, as well as average Soviet citizens. While a considerable proportion of the labour force came to the construction on their own, inspired by the industrialisation drive, thousands of repressed *kulaks* ^71^ and dozens of prisoner specialists were brought to the construction site by force. While the predominant majority of the labour force at *Magnitostroy* were ethnic Russians, the presence of Tatars, Bashkirs, Kazakhs and Ukrainians suggested an influx of labour force from the neighbouring regions and Soviet republics. By the end of the construction period in the late 1930s, Magnitogorsk had become not just a steel city, but, similar to the BAM, a microcosm of the Soviet Union.

Infrastructure projects in other regions of the USSR had the same “civilizing” mission, as the northern one, in their efforts to “bring culture” to “backward” peoples and build the nation. For example, building socialism in the case of the Turkestan-Siberian Railroad (Turksib) in 1926–1931 implied the “ending [of] the exploitation of such small nations as Kazakhs, which in turn meant supplying them with the economic, educational and cultural infrastructure of ‘modern’ nations.”^72^ Planned for strategic and economic reasons (transportation of cotton and corns), the Turkestan-Siberian Railroad brought to the Kazakh steppe not only trains, but also a cultural revolution. In comparison to Magnitogorsk, this project relied mostly on the local labour force, and, to a lesser extent, on migrants from the neighbouring regions.

## Developing and studying northern infrastructure in post-Soviet Russia

Since the dissolution of the USSR, the role of the Russian state in the Arctic has shifted in parallel to the changing political climate – from the withdrawal from significant investments in the 1990s to the new militarisation plans in the current decade. At the same time, Russia’s resource politics and economic interests, focused on hydrocarbons’ extraction, have provided a field for the emergence of new stakeholders in the Arctic in the post-Soviet period.

The current presence of the state in the Arctic and the North is regulated by several fundamental federal laws, including national security strategies^73^ and a series of regional socio-economic development programs.^74^ These documents foresee the modernisation and establishment of infrastructures that should foster international trade, promote environmental safety and the quality of life of the local population. The development of northern transportation infrastructures is further reinforced in the national transportation strategies.^75^

The extensive network of modern transportation and industrial infrastructures established in the northern regions in the Soviet period has been degrading due to the shrinking state investments. Cancelled air connections, malfunctioning ground transportation, degrading sea ports became the everyday life realities that local residents and travelers had to face in the North in the period of perestroika and the following socio-economic crisis*.* Abandoned roads and railroad tracks, collapsing electricity transmission lines, unfinished industrial and apartment buildings are still features of modern land- and cityscapes in the Russian North (e.g. in towns and settlements along the Baikal-Amur Mainline in East Siberia).

Russia’s plans for the (re)construction of infrastructure objects are presently fuelled by a (re)militarisation campaign and a continued drive for large-scale resource extraction. A number of economic and sociological studies demonstrate that the development of transportation and communication networks has a growing geopolitical importance for supporting the state’s presence and facilitating the flows of resources and goods in the Arctic and the North. These studies remind us that the Russian Arctic has been a region of dislocation of a series of battalions, radar stations, anti-aircraft interceptors and the Northern Fleet. In the context of the growing tensions over the domination between the arctic states, these military objects with their adjacent on-ground and coastal transportation infrastructures, will most likely be attracting state investments in the years to come.^76^ At the same time, the fact that up to 80% of the oil and 90% of the gas in Russia are extracted in the North indicates the significance of transportation infrastructure (railroads, pipelines and marine terminals) to supply these key resources on which the domestic economy heavily depends.^77^

The logistic research on infrastructure suggests strategies for establishment of the backbone transportation network in Russia’s Arctic and Subarctic regions. Such strategies foresee an increased role of the state in the establishment of latitudinal and longitudinal corridors (e.g. roads and railroads), leading to the key hubs (e.g. sea ports), coordinating the work of all types of transport. In this integrated network, the Northern Sea Route/North East Passage and a number of existing (Transsiberian, Baikal-Amur) and projected (Transpolar, Transpacific) railroads should play key roles.^78^ Further logistic studies offer spatial models for the establishment of mobile (temporary) settlements and innovative technological solutions for the modernisation of railroad and seaport infrastructures in the Arctic.^79^

In comparison to socio-economic studies and development strategies, demographic research looks more into migration processes, population change and larger-scale social dynamics, boosted by industrialisation and infrastructure build up. With the transition from the central planning to the decentralisation of finance, the population size of the Russian North became a burden for the state. The new labour resource policy is driven mostly by private companies, which take the costs of training and recruiting young skilled workers in resource extraction regions. State programs running parallel to it aim at the resettlement of pensioned, disabled and nonworking people from the North. These trends result in the growing proportion of temporary population, usually shift-workers, recruited by construction and extractive companies operating in the North.^80^ The current disparities in the migration patterns lead to the emergence of “growth poles” in the expanding resource extraction provinces (Khanty-Mansiyskiy Okrug, Yamalo-Nenetskiy Okrug), and “ghost towns” as well as “non-promising settlements” in remote regions of Siberia and the Far East (Buryatiya, Zabaykal’skiy Kray, Magadan, Chukotka, etc.) with high living and industrialisation costs. The majority of the incoming population views their stay in the North as temporary: it is rather the financial stimuli than ideological factors that attract them to stay.^81^

New industrial infrastructures, currently built in the Russian North, are mostly associated with the activities of extraction companies. While some companies expect federal and regional investments into the (re)construction of electricity lines and the transportation network, building and maintaining rigs, processing plants, local roads and side tracks leading to the mines and oil fields, clearly lies within the sphere of private responsibility. According to international standards and the Russian federal legislation,^82^ environmental impact assessments are a necessary prerequisite for the launch of the companies’ operations. A comprehensive legal tool for assessing social impacts of extraction activities in Russia is still missing. Yet, such assessments go along with the environmental issues within the wider concept of corporate social responsibility, especially in the case of large-scale companies, which care about their reputation.^83^

This situation gave rise to a number of applied social and interdisciplinary studies of industrial and transportation infrastructures, assessing risks and benefits of projected pipelines,^84^ railroads,^85^ and infrastructure modernisation programs.^86^ In Russia, this kind of research has become an important agenda for the field of applied legal anthropology. It focuses on the rights of local communities, particularly, indigenous peoples of the North, their traditional lifestyles and activities, as well as their resource use practices vis-à-vis industrial development, within the frameworks of indigenous rights, customary law and legal pluralism.^87^

Case studies of infrastructures in the Russian North are addressed in the context of industrialisation, sustainable development and climate change within the international RATIC initiative. The working group report, presenting case studies on several circumpolar regions with intensive industrial development, also covered two regions of the Russian North – the Yamal Peninsula, where the effects of climate change and oil development are overlapping, and the Arctic industrial city of Noril’sk, where permafrost changes damage the buildings, reconfiguring the cityscape. RATIC general recommendations call for more attention to Arctic socio-ecological systems, elaboration of new tools for monitoring infrastructure and landscape changes as well as coordination of international research efforts.^88^

## Case studies from the Russian North

The two case studies below represent different periods of Soviet modernisation and infrastructure build-up, but share a number of striking similarities concerning ideologies, human resource management and construction history. This continuity explains the vitality and applicability of the term “communist construction site” widely used in relation to large-scale infrastructure projects throughout the Soviet period (see above). Those infrastructures built in the extreme conditions of the arctic and subarctic environment were not only grandiose technological endeavours symbolising human domination of “nature”, but also social engineering and identity building projects. The current modernisation efforts prove the continued significance of these objects for the state and other stakeholders and demonstrate the entanglements of humans with their built environment.

### The Baikal-Amur Mainline (BAM)

The example of the Baikal-Amur Mainline (BAM) tells a story of many superlatives. First and foremost, it was one of the largest Soviet development projects, which involved the construction of a railroad and an extended network of train stations, settlements and cities and entailed a large-scale population change.

Yet, the history of the BAM begins long before the Soviet era, when the idea of a new railroad line running north of Lake Baikal, parallel to the Transsiberian Railroad (Transsib) arose. In the late XIX century, first construction projects for such a railroad were discussed^89^ motivated by plans for natural resource extraction and economic development of East Siberia and the Russian Far East.^90^ The immediate predecessor to the contemporary railroad was the BAM section stretching from Komsomolsk-na-Amure to Sovetskaya Gavan’ in Khabarovskiy Kray built between 1932 and 1953 by BAMLag camp inmates, military personnel and prisoners of war. That project was abandoned after Stalin’s death in 1953, and the idea of restarting the BAM construction gained official favour only in the Brezhnev era, nearly two decades later.

The late socialist BAM represented the last megalomaniac communist industrial project and “the government’s attempt to exploit the USSR’s vast natural resources for propagandistic and economic reasons”.^91^ Moscow hoped that a completed BAM would bolster collective faith in the command-administrative system and serve as the prototype for further conquests of the Soviet Union’s vast and resource-rich northeastern frontier in the twenty-first century.^92^

In 1974, the Communist Party’s youth organisation announced the beginning of BAM construction and a labour mobilisation campaign under Soviet propaganda slogans. In total, about 500,000 BAM builders were lured to the region from all over the Soviet Union. The majority of the incoming labour force was represented by young educated men, who often intermarried with female BAM builders or local women and settled down in the towns and settlements built by and for the so-called BAM builders *(bamovtsy)* along the railroad.^93^ The BAM project became a population magnet and a testing ground for Soviet ethnic policies. It drew labour migrants with diverse cultural backgrounds who have formed the majority of today’s population of the region.

The official BAM’s leitmotiv was that “the whole nation is building the BAM!”.^94^ Indeed, the BAM builders came from all over the Soviet Union. Thus, the project was not only about the creation of infrastructure but also about the consolidation of a (socialist) nation, as well as about building communism. While Soviet era publications stressed the solidarity among *bamovtsy* ^95^ some recent studies of the BAM take a more critical perspective on Soviet identity policies and its practices regarding builders.^96^ Although the BAM project was multi-national, the so-called working brigades were frequently formed based on their place of origin (usually, a region or a republic), and were commissioned to build a particular railway station and/or a settlement.

Until today, the built environment along the BAM reflects the spatial distribution of workers during the construction period. The designs of stations and adjacent buildings point to the ethnic and geographic background of the building brigades, such as the station in Novaya Chara, which was built by workers from the Kazakh Socialist Soviet Republic (SSR). Another example is the small settlement Niya, which was constructed by a brigade from the Georgian SSR.^97^ Until today, ornaments, place names and a memorial remind of Georgians working on the BAM in the past.

While Moscow was the primary locality of conceptualising the BAM, Tynda – once a tiny settlement of gold miners amidst the vastness of the boreal forest – became its northern counterpart. Therefore, it is no surprise that the city of Tynda was conceptualised as a copy of Moscow.^98^ Due to its strategic position at the junction of the BAM and its northern extension leading to the Sakha Republic (Yakutiya),^99^ Tynda quickly became the “capital” of the BAM region (by the late 1970s) and a regional centre for planning and construction.

The BAM construction boom was followed by an economic bust and public criticism of the project during the 1990s. While the major parts of the mainline were built between 1972 and 1984, some sections, such as the branch to the North, are still under construction. Currently, the BAM is operated by the Rossiyskie Zheleznye Dorogi (RZhD), Russia’s largest railroad company owned by the state. The main branch of the present-day BAM is approximately 4300 km long, while its northern extension – the Amur-Yakutsk Mainline (AYaM) runs over 1000 km. The BAM and its branches cross the northern districts of six federal subjects – Irkutskaya Oblast’, Buryatiya, Zabaykal’skiy Kray (all in Eastern Siberia), as well as the Republic of Sakha (Yakutiya), Amurskaya Oblast’, and Khabarovskiy Kray in the Russian Far East.

The largest cities of the BAM include Komsomol’sk-na-Amure (263,906 people), Neryungri (61,747 people), Tynda (36,275 people) and Tayshet (35,485 people).^100^ However, the typical BAM settlement is a town of a smaller scale, with the population ranging from 200 to 3000 residents.^101^ The population size of BAM communities peaked in the mid-1980s and rapidly declined during the socioeconomic crisis and mass exodus of the population from the North in the 1990s. Currently, the BAM region continues to lose its residents, in line with the annual, well-documented out-migration from Siberia and the Far East.^102^ The permanent population is steadily being replaced by industrial shift workers from neighbouring regions and other parts of Russia, as well as by seasonal agricultural workers and traders coming mostly from Central Asia, the Caucasus and China.

With its existing branches connecting remote settlements with administrative centres (and projected side lines leading to mineral deposits), the railroad provides a reliable transportation network for people, goods and resources, although the latter were certainly the driving force in making the project happen. The state plans for the establishment of industrial clusters, including mining and processing plants and transportation infrastructures, approved back in the 1980s, are being revised and modified into attractive investment projects, some of which have already found their developers, while others are still waiting for a new economic boom.

In 2014, the programme for modernisation and construction of the second track of the mainline was launched. The programme is clearly aimed at increasing the BAM’s cargo transportation capacity, while passenger services have been decreasing. With the introduction of inconvenient schedules and cancellation of commuter trains, the state has imposed a particular form of infrastructural violence^103^ on local residents often dependent on the railroad. It remains unclear how the railroad modernisation and increased resource extraction will benefit the communities along the BAM, since the revenues of extraction companies flow primarily to the federal budget. Currently, local residents relate to the railroad rather as a symbol of the socialist construction period and an infrastructure full of promises and expectations^104^ than a means of passenger transportation or a source of sustainable community development.

It is not surprising that the “construction site of the century”, as the project was promoted by the Soviet state in the 1970s, has triggered an avalanche of studies. A particularly productive genre has been historiography, providing critical studies of this Brezhnev-era infrastructure project.^105^ Previous social research of the BAM region has focused on demographic change and migrations,^106^ geographic and social mobility,^107^ with few ethnographic case studies of the BAM’s impacts on indigenous communities.^108^ More recent sociological publications on memories of the BAM builders, show a renewed interest in the topic.^109^

The project CoRe (“Configurations of Remoteness”)^110^ uses the BAM as a testing case study to highlight the entanglements of the local communities with the railroad technosocial meshworks under conditions of remoteness and resource extraction booms. This project, launched in 2015, has yielded the first findings and publications. It breaks new ground as a contribution to the severely understudied field of transportation infrastructure and built environment in the circumpolar North, as suggested in the introduction. The concept of “remoteness” offers the opportunity to assess social effects of transportation networks on non-central, densely populated and presumably disconnected communities in sparsely populated regions more readily by comparing the BAM region with other *resource frontiers*. The concept and ethos of the “frontier”,^111^ as well as Soviet industrial policies were the driving forces behind the construction of the BAM mono-industrial towns. Current dependence on hydrocarbon extraction and degradation of infrastructures due to shrinking state investments renders these local communities more remote in the sense of both political imaginary, economic development and living standards.^112^

Another central notion of the project – technosocial meshworks, refers to the abovementioned work by Ito and Okabe, on the one hand, and to Ingold’s “meshwork of interwoven lines” as an alternative to a more structured network, on the other. Overlapping social and technological networks form transportation hubs as spaces of communication, negotiations and exchange.^113^ Technosocial networks of the BAM include both a system of infrastructure, including cities, towns and stations as well as the network of social relations connecting the railroad settlements and indigenous villages off the road. Larger BAM communities, which often play a role as administrative centres, serve as the centres of attraction for rural (mostly, indigenous) migrants, as well as the major transportation and communication hubs, where intense intercultural contacts, administrative procedures and stakeholders’ negotiations on development projects occur.^114^

The research shows that historically shaped entanglements of indigenous and migrant populations with the railroad are still influencing social dynamics and identity building processes today. The former builders in the region usually still identify themselves as *bamovtsy.* Currently, more than 40 years after the opening of the main parts of the BAM, they share a similar ideology, emotionally charged memories and experiences. Those are often conveyed to the next generation, some of whom call themselves “children of the BAM”. In rare cases, *bamovtsy* identity was, at least, situationally, adopted by indigenous Evenki individuals who were involved in the construction process.

The BAM’s agency is reflected both in the natural and built environments. For example, the railroad has considerably changed indigenous Evenki landscapes. Herders and hunters now have to find new routes due to the destruction and pollution of their grounds and pastures. On the other hand, they have integrated the railroad into their food supply and mobility (between villages and *taiga* camps) schemes.

Many shanty temporary settlements, initially erected only for the period of railroad construction, remain occupied. This is for example the case with the settlements “Solnechnyy” and “Sinegor’e” in Aldanskiy Kray, where old structures, as well as place names, recall the BAM’s past. However, the mentioned settlements are inhabited partly by former BAM builders and their descendants, but mostly by incoming migrants, who do not relate to the BAM history anymore.^115^ The case of the railroad stations Tommot and Aldan, both opened in the early 2000s in the Sakha Republic demonstrates that architectural design continues to serve as a platform for showcasing national and ethnic identity politics. A Yakutsk based architect explained, that Aldan with its predominantly Slavic population received a train station in a classical style, whereas the design of the Tommot station resembles the indigenous Sakha summerhouse *uraha.* ^116^ Interestingly, some local Evenki consider the station to resemble Evenki huts and not Sakha dwellings.^117^

The BAM as a means of transportation has been enabling the movement of different kinds of matter. Most importantly, since its very conception, the BAM has served the transportation of cargo and currently plays an increasing role in supply of natural resources (ore, coals, timber, oil) to the Asian markets. Its importance for the passenger connection, however, has been changing more unpredictably. Thus, in recent years, passenger commuter trains, which have served a reliable means of transportation between local communities, are being cancelled. Considering the dependence of the population – from former BAM builders to Evenki reindeer herders – on trains, this cancellation policy became a form of infrastructure violence, the notion explained above. At the same time, preliminary results of the project’s mobility survey, aimed at local residents, showed that the role of the BAM as a social phenomenon – an agent of population change and a source of shared life and family histories – is much bigger than that of the transportation. In fact, people prefer personal cars and other public transport, wherever it is available, for traveling at shorter distances.

Studying the role of the BAM in contemporary mobility regimes is one of the goals of the geographic component of the CoRe project. It combines ethnographic fieldwork and qualitative GIS mapping with the method of cartographic storytelling.^118^ The qualitative (field notes, interviews, narratives, mind maps, photographs, etc.) and quantitative data (spatially explicit demographic and socio-economic data, survey data, etc.) will be integrated in order to track and visualise the meshworks of lived entanglements of people and infrastructure for analytical and dissemination purposes.

### Northern Sea Route

The second case study demonstrates another example of a large-scale transportation infrastructure, but with a much longer history of exploration, building and use. The Northern Sea Route (NSR) is an arctic shipping route and, in a more complex sense, a network of seaports and settlements stretching along the coastal areas from the Novaya Zemlya in the west to the Bering Strait in the east. The Russian definitions of the NSR or NSR Area with their officially demarcated borders include marine infrastructures in six federal subjects adjacent to the Arctic Ocean.

This transit route lying north of Russia and linking the North Atlantic and the North Pacific oceans was historically referred to as the North-East Passage (NEP), the term still used internationally as an equivalent to NSR. These competing terminologies reflect, on the one hand, the long international history of exploration and use of the passage, and, on the other hand, the Soviet period, when this transportation corridor was hardly accessible for international users, as well as Russian surveillance over the route at present day.

The first practical demonstration that the Arctic Ocean and the North Pacific are connected was conducted in 1648 by Semen Dezhnev. Vitus Bering, whose name is now tied to the strait connecting these two oceans, commenced scientific studies of this connection in the eighteenth century, continued by Russian, American and Norwegian expeditions under the command of Ferdinand Wrangel, George de Long and Fridtjof Nansen respectively in the nineteenth century. Adolf Erik Nordenskiöld was the first to make the full Northeast Passage in 1878–1879.

The NSR history of the twentieth century, from both socio-economic and technological perspectives, includes four periods: (1) beginning of the international commercial shipping until the 1930s; (2) construction of the Arctic fleet and the ports between the 1930 and 1950s and the spread of the term Northern Sea Route in Soviet official discourse; (3) regular seasonal (summer and autumn) use of the route from the 1950 to the 1970s; (4) year-round exploitation of the NSR since the 1970s.^119^

Since the Stalin period, the NSR became a domestic transportation route closed for international users. The establishment of the NSR Administration *Glavnoe Upravlenie Severnogo Morskogo Puti (*abbreviated as “Glavsevmorput’”) in 1932 was a milestone in the NSR history. The body controlled the territory over 2 million square kilometres and coordinated academic explorations, resource extraction, shipbuilding and transportation. Most importantly, it managed the human resources, including indigenous population and arriving forced labour working in cooperation with GULAG. In 1938, the many functions of “Glavsevmorput’” were transferred to different ministries. Yet, large-scale scientific exploration and industrialisation efforts, undertaken in this period, engendered the myth of the “Red Arctic”, which is still living in Russian polar cities.^120^ This myth, standing for glorious human achievements in the Arctic, relates back to the Soviet development policies and ideologies of “mastering the North” for the strategic and resource extraction purposes.

The NSR’s importance for domestic supply of goods, foods and raw materials reached its peak in the 1980s. With the curtailing of state subsidies and the delegation of the responsibilities for the maintenance of infrastructure in the regions during the 1990s, the route’s exploitation became unprofitable. In the period of the socio-economic crisis, the volume of shipments dramatically declined and the seaport infrastructure started degrading. While the largest ports in the Arctic cities of Arkhangelsk, Murmansk and Norilsk have developed urban infrastructures, smaller communities (e.g. Dikson, Pevek, Provideniya) suffer tremendous population loss and request urgent infrastructural modernisation. At the same time, the emergence of new ports (e.g. Sabeta on the Yamal Peninsula constructed for the export of liquefied natural gas) is connected with the current oil and gas extraction projects.

The opening of the NSR to international traffic in 1991, along with environmental changes leading to less sea ice and, thus, facilitating year-round shipping, has attracted the attention of international companies and other stakeholders. During the period 1993–1999, the International Northern Sea Route Programme, an interdisciplinary research initiative, investigating the status quo and potential impact of international shipping, was implemented. This research, has shown that the route plays a key role for community development and domestic supply within the local transportation networks. It is crucially important for its eastern sector (i.e. Chukotka) and plays a significant role for its “central” segments (Yakutiya and Krasnoyarskiy Kray), where alternative means of transportations, such as rivers and the Transsiberian Railway, are available. Finally, it boosts economic development in the booming oil extraction regions (Nenets and Yamalo-Nenets Districts) in its western segment. It also showed that potential NSR cargo flows of non-ferrous metals and ores, timber, coal and deliveries to Arctic settlements, are likely to remain stable.^121^ At the same time, despite the optimism regarding the potential of NSR and other Arctic routes in commercial shipping as an alternative to the Suez Canal is overstated. Jurisdictional disputes, environmental as well as infrastructural challenges, such as shallow waters, lack of modern deep-water ports, as well as search and rescue capabilities lessen the economic viability of the route.^122^

The recently increased public attention to NSR, fuelled by its modernisation programme, calls for revisiting its history and raises a number of new research questions. How has the NSR project been constructed and imagined in different times and at different scales? What are the experienced and potential impacts of shipping on the infrastructure of sea ports and settlements along the route? Which place do NSR discourses and concepts play in state and local identity building processes? These and other questions, some of which are addressed within a recently supported project on NSR,^123^ provide new materials and grounds for research in the field of anthropology of infrastructure and built environment in the Arctic.

## Conclusions

The case studies presented above focus on Soviet and Russian experiences. Our rationale is not to highlight how different the Russian situation was and is but to use Russian experiences as vivid examples of more wide-spread tendencies. One case in point is the role of the state. While many large-scale building and infrastructure projects in various parts of the world depend on investments and/or political and logistical assistance by governments, this seems to be even more relevant for the polar regions. Given the political and legal status of Antarctica, the almost exclusive agency of states in the southern polar regions is not surprising.^124^ In the Arctic and Antarctic, we see differences that run to a certain degree parallel to political and ideological traditions. While the Soviet Union was entirely state-centred (at least, since the 1930s), and the Russian Federation increasingly so again in recent years, Alaska and northern Canada seem to represent the other end of the spectrum. Still, even the large-scale building and infrastructure projects of northern North America are difficult to imagine without government input. This is true for the historical (military) project of the Alaska-Canada Highway, as well as for the Canadian High Arctic Research Station (CHARS) in Cambridge Bay, Nunavut, scheduled to be opened in summer 2017. Even the Trans-Alaska Pipeline System (TAPS) completed in 1977, while funded through a consortium of oil producers, would have been impossible without assistance from the U.S. federal government (among other things, it consisted of providing legal protection for investments by settling land rights issues through the Alaska Native Claims Settlement Act).

Thus, the question is whether our two case studies of northern transportation infrastructure projects point to “polar” specifics. One such specific could be that – under conditions of low population density and a relative paucity of lines of transportation and information, that is conditions typical of polar regions – new additions to communication networks (such as building the BAM or developing the NSR) have social consequences that might be more profound – but certainly are more visible – than in more infrastructure-rich parts of the world. Another dimension of specificity might be given through the extreme environmental conditions in the Arctic and Antarctic. These make the maintenance of existing and the construction of new infrastructure more demanding, in terms of both financial and human resources, than elsewhere. This is another reason for the above-mentioned state-centeredness of polar infrastructures.

This article argues for more attention to the built environment of the polar regions. While, as anthropologists, we are particularly interested in the “social lives” of the built (and in the “infra” in infrastructure), the materiality and knowledge production processes behind it are no less relevant for a contemporary perspective. We thus encourage a multitude of inquiries into the human/built environment relations, whether they are based on historical, ecological, social or science studies perspectives. The ethnographic dimension is relevant in every case, no matter whether the scales of attention are local, global or in-between.^125^

The intention of this contribution is of course not to deny the obvious, namely that many northern regions are among the least densely populated regions of the globe and, thus, environmental modifications by humans are less prevalent than in urban centres. On the contrary, the relative absence of the built has been the starting point for two arguments. On the one hand, it is important for us to note that relative absence is not equal to absolute absence. In other words, it is important to highlight the agency of the built environment in polar regions because it could be more easily overlooked here than in other regions. On the other hand, while residents of New York City or other megacities might spend most of their days in environments entirely devoid of any “natural” traces, people residing in villages and towns of the Arctic and Subarctic often list their “closeness to nature”^126^ as defining attractive dimensions of their lives. Thus, part of the relevance of studies into the built environment in “remote” regions is that the interwovenness of the “natural” and the “built” is both more visible and invisible. It is visible in that most residents in the circumpolar North in the twenty-first century (and for quite some time before that) rely on high-tech tools, facilities and infrastructures in order to live “close to nature”. Invisibility then is a product of the juxtaposition between the urban (and non-arctic) and the “natural” in polar contexts. We believe that the specific constellations of natural/built hybrids in the Arctic and Antarctic deserve particular attention because they demonstrate that these two analytical terms cannot be separated. Despite our use of the analytical distinction natural/built, we largely adopt an Ingoldian dwelling perspective that highlights that humans make use of the affordances of (natural and built) environments, and have to deal with the agency of these environments.^127^

Despite our focus on the twentieth and twenty-first centuries, we maintain that the built environment is not an invention of modernity. Likewise, our position that human habitation and the built environment are indivisibly linked, makes it clear that a focus on the built does not equal a focus on non-indigenous groups of people. Still, it might make sense to state a difference as a rather generalising observation. While all contemporary residents of polar regions – indigenous and non-indigenous alike – live in built environments that include a large dose of outside technologies, indigenous peoples in the Arctic (and elsewhere in the world) have a long historical track record of living in built environments based on local and (mostly) self-reliant technologies. Thus, while there is no absolute difference between industrial and pre-industrial built environments, it might make sense to distinguish large-scale endeavours of recent centuries from pre-colonial building activities. If nothing else, the spatial and social distance between builders and built seems to characterise colonial legacies in polar regions up to the present day.

Finally, and seemingly contradicting the lines of argumentation developed above, what about the entanglements between built and natural environments? While we have focused on infrastructure and the built environment, we see good reasons for eventually moving beyond the analytical distinction (of built vs. natural) advocated for in our contribution. Where Thomas Hughes was still arguing for the primacy of what he calls the “human-built world”,^128^ we have come to understand that behind the façade of “human-built” are many non-human dimensions, thereby calling for the incorporation of more-than-human^129^ and actor-network theory^130^ approaches. While Arctic and Antarctic regions are ideal testing grounds for such integrated approaches, we need a better understanding of the social lives of polar infrastructure and altered environments, be they based on human building activities, permafrost thawing or crumbling concrete. On the one hand, this means the acknowledgement of the interaction and entanglement of the “built” and the “natural”. On the other hand, while our call to move “beyond wilderness” is an attempt to highlight human activity in polar regions, a call to think “beyond the built” might be more appropriate in urban spaces. After all, understanding the significance of the Anthropocene should not be limited to the acknowledgement of humans as a geological force but should include the entanglements and feedbacks of anthropogenic and non-anthropogenic drivers of change.

## Funding

This work was supported by the Austrian Science Fund [grant number P27625-G22].

## Disclosure statement

No potential conflict of interest was reported by the authors.

## Footnotes

^1^ See, e.g. Gómez-Pompa and Kaus, “Taming the Wilderness Myth”; and Grant, “Arctic Wilderness”.

^2^ See Tin, Summerson, and Yang, “Wilderness or Pure Land”, for a recent example.

^3^ Behrends, Reyna, and Schlee, *Crude Domination*.

^4^ Wilson, “Est’ Zakon, est’ i zvoi Zakony”; Novikova, *Okhotniki i neftianniki*; Saxinger, *Unterwegs*; and Stammler and Peskov, “Building a ‘Culture of Dialogue’”.

^5^ Larkin, “The Politics and Poetics of Infrastructure”.

^6^ Star, “The Ethnography of Infrastructure”.

^7^ See, e.g. Harvey and Knox, *Roads*; Nielsen and Pedersen, “Infrastructural Involutions”; and Anand, “Leaky States”.

^8^ See, e.g. Humphrey, “Ideology in Infrastructure”; and Collier, *Post*-*Soviet Social*.

^9^ Kaschuba, *Die Überwindung der Distanz*; and Schivelbusch, *Geschichte der Eisenbahnreise*.

^10^ Star, “The Ethnography of Infrastructure,” 380.

^11^ Larkin, “The Politics and Poetics of Infrastructure,” 329.

^12^ Rodgers and O’Neill, “Infrastructural Violence”.

^13^ Ito and Okabe**, “**Technosocial Situations”.

^14^ Fisch, *On the Train*; and Fisch, “Tokyo’s Commuter Train Suicides”*.*

^15^ Carse, “Keyword: Infrastructure,” 29.

^16^ Ibid., 35.

^17^ Ibid.

^18^ Infrastructure cannot be limited to the physical, though; for a recent example of the importance of technical standards on the effective power of infrastructure, see Carse, “Toward a Political Ecology of Infrastructure Standards”.

^19^ For a classical anthropological definition of the built environment see Lawrence and Low, “The Built Environment and Spatial Form,” 454: “The built environment is an abstract concept employed here and in some of the literature to describe the products of human building activity. It refers in the broadest sense to any physical alteration of the natural environment, from hearths to cities, through construction by humans”.

^20^ See, e.g. Ingold, *The Perception of the Environment*.

^21^ Ibid., 172–88.

^22^ Rapoport, “Spatial Organization and the Built Environment,” 465.

^23^ Ibid., 464.

^24^ See, e.g. Lawrence and Low, “The Built Environment and Spatial Form”; and Low, “The Anthropology of Cities”.

^25^ Humphrey, “No Place Like Home in Anthropology,” 16.

^26^ See, e.g. van der Hoorn, “Indispensable Eyesores”; and Sparkes and Howell, *The House in Southeast Asia*.

^27^ Buchli, *An Anthropology of Architecture.*

^28^ See, e.g. Bourdieu, “The Kabyle House or the World Reversed”; and Humphrey and Vitebsky, *Sacred Architecture*.

^29^ Ingold, *Making.*

^30^ Humphrey, “Ideology in Infrastructure,” 39, 55; and Buchli, *An Archeology of Socialism.*

^31^ Rapoport, “Spatial Organization and the Built Environment,” 496.

^32^ Maudlin and Vellinga, *Consuming Architecture.*

^33^ Lawrence and Low, “The Built Environment and Spatial Form,” 456.

^34^ See, e.g. Povoroznyuk and Funk, “Urbanizatsiya i korennye narody Severa”; and Schweitzer, “Korennye narody i urbanizatsiya”.

^35^ This situation has started to change and the “urban Arctic” is finally receiving more attention (see, e.g. Orttung, *Sustaining Russia’s Arctic Cities*, as just one recent example).

^36^ A lot of the infrastructure literature on the Russian North has focused on roads, outside and between urban areas (see Argounova-Low and Prisyazhnyi, “Biography of a Road”, for a recent example).

^37^ Hacquebord, *LASHIPA*.

^38^ Hacquebord, “The History of Exploration,” 131.

^39^ Ibid., 132.

^40^ See, e.g. Kraikovski et al., “The Organization of Pomor Hunting Expeditions”; and Kraikovski, “Productivity and Profitability of Russian Spitsbergen”.

^41^ See, e.g. Hackett et al., *The Handbook of Science and Technology Studies*.

^42^ See, e.g. Dey Nuttall, “Organizing Polar Science”.

^43^ See, e.g. Doel, Harper, and Heymann, *Exploring Greenland*.

^44^ Schweitzer, “Can One See the Arctic”.

^45^ Doel, Wråkberg, and Zeller, “Science, Environment, and the New Arctic,” 2–3.

^46^ Ruiz, “Arctic Infrastructures”.

^47^ Ibid., 20.

^48^ Salazar, “Polar Infrastructures”.

^49^ Scott, *Seeing Like a State*.

^50^ Ibid., 88–90.

^51^ Ibid., 345–6.

^52^ The notion Krayniy Sever (literary, “Extreme North”) was introduced to highlight climatic conditions (low temperatures, sharp fluctuations in weather, permafrost landscapes, etc.). Nowadays, the concept of *Krainiy Sever* (rendered in English as “Far North”) denotes a number of environmental, socio-economic and infrastructural characteristics of arctic and subarctic territories and is widely used in official discourses, documents and state programmes.

^53^ Slavin, *Osvoenie Severa*, 11–15.

^54^ Bolotova, *Conquering Nature*, 30.

^55^ Ibid., 45–8.

^56^ Josephson et al., *An Environmental History of Russia*, 7.

^57^ Bolotova, *Conquering Nature,* 73.

^58^ Josephson et al., *An Environmental History of Russia*, 8–11.

^59^ Josephson, “‘Projects of the Century’,” 519.

^60^ Marks, *The Road to Power: Transsiberian Railway*, 10.

^61^ Grant, *In the Soviet House of Culture*.

^62^ Fondahl, *Gaining Ground*; Habeck, “Learning to Be Seated”; and Holzlehner, “Engineering Socialism”.

^63^ Spevakovskiy, “Traditsionnoe v sovremennoy khozyaystvennoy deyatelnosti,” 121; and Habeck, “Learning to Be Seated,” 155–79.

^64^ Kotkin, *Magnetic Mountain*, 20.

^65^ Rozhanskiy, *Sibir’ kak prostranstvo pamyati*.

^66^ Thomas, “On Guard at BAMlag,” 3. Also see the case study below.

^67^ Graham, *The Ghost of the Executed Engineer*, 6.

^68^ Engel, “Public Space in the ‘Blue Cities’ of Russia”.

^69^ Kotkin, *Magnetic Mountain*, 33.

^70^ Graham, *The Ghost of the Executed Engineer,* 59.

^71^ A term widely used in historical and anthropological literature to refer to the peasantry, resisting to Soviet collectivisation campaign and/or repressed by the Soviet regime (especially, during the Stalin’s period of rule).

^72^ Payne, *Stalin’s Railroad*, 9–10.

^73^ President of the Russian Federation, “Osnovy gosudarstvennoy politiki”; Government of the Russian Federation, “Strategiya razvitiya Arkticheskoy zony”; and Government of the Russian Federation, “Sotsial’no-ekonomicheskoe razvitie”.

^74^ Government of the Russian Federation, “Strategiya sotsial’no-ekonomicheskogo razvitiya”.

^75^ Government of the Russian Federation, “Transportnaya strategiya Rossiyskoy Federatsii”, “Strategiya razvitiya zheleznodorozhnogo transporta”.

^76^ Frolov, “Development of the Russian Arctic Zone,” 561.

^77^ Selin and Bashmakova, “Znachenie severnykh i arkticheskikh regionov”.

^78^ Adrianov, “Formirovanie transportnoy infrastruktury”.

^79^ Litovsky, “Kontseptsiya razmeshcheniya v Arktike”.

^80^ Saxinger, “Lured by Oil and Gas”.

^81^ Heleniak, “Growth Poles,” 134, 146–52.

^82^ Government of the Russian Federation, “Ob ekologicheskoy ekspertize”.

^83^ Tishkov, ed., *Rossiyskaya Arktika*, 36.

^84^ Sirina and Fondahl, “Evenki severnogo Pribaykal’ya”.

^85^ Bogdanov, Golovatin and Morozova, “Ekologicheskie riski”.

^86^ Meschtyb, Forbes, and Kankaanpää, “Social Impact Assessment”.

^87^ Novikova, *Olen’ vsegda prav*.

^88^ Walker et al., *Rapid Transitions*.

^89^ Yurgenson, “Istoria issledovaniya,” 23–7.

^90^ Grützmacher, *Die Baikal*-*Amur*-*Magistrale, 308*.

^91^ Ward, *Brezhnev’s Folly,* 2*.*

^92^ Ibid.*,* 2–5.

^93^ Belkin and Sheregi, *Formirovanie naseleniya v zone BAM*.

^94^ Ward, *Brezhnev’s Folly*, 109.

^95^ See, for example, Argudiaeva, *Trud i byt molodezhi BAMa.*

^96^ Ward, *Brezhnev’s Folly.*

^97^ Röhr, “Poselok Niya (Gruzinskaja) v gody Gruzstroybama”.

^98^ Ibid.

^99^ Grützmacher, *Die Baikal*-*Amur*-*Magistrale*, 311.

^100^ Vserossiyskaya perepis‘ naseleniya 2010.

^101^ Administration of Tyndinskiy Rayon, *Itogi sotsial’no*-*ekonomicheskogo razvitiya*.

^102^ Statistical Bureau of Amurskaya Oblast’, *Sotsial’no*-*ekonomicheskoe polozhenie.*

^103^ Rodgers and O’Neill, “Infrastructural Violence”.

^104^ Compare: Harvey and Knox, *Roads.*

^105^ Grützmacher, *Die Baikal*-*Amur*-*Magistrale*; *Röhr, Die Bajkal*-*Amur*-*Magistrale*; Ward, *Brezhnev’s Folly.*

^106^ Bulaev, *Etno*-*natsional’nye osobennosti,* Argudyaeva, *Trud i byt molodezhi BAMa.*

^107^ Karpov, *Problemy sotsial’noy mobil’nosti.*

^108^ Boiko, *Opyt sotsiologicheskogo issledovaniya*; Vinokurov, “Evenki”.

^109^ Bogdanova, “Kak utopiya stala real’nost’yu”.

^110^ “Configurations of Remoteness (CoRe): Entanglements of Humans and Transportation Infrastructure in the Baikal-Amur Mainline (BAM) Region” (funded by the Austrian Science Fund (FWF) [P27625-G22]).

^111^ Tsing, “Natural Resources, Capitalist Frontiers”.

^112^ Povoroznyuk, “Social Dynamics and Sustainability”.

^113^ See also Kleinman, “Adventures in Infrastructure,” for a case study on the use of spaces of public transit for social network building and communication.

^114^ Povoroznyuk, “Gorodskie Aborigeny BAMa”.

^115^ S. Schiesser, field diary, April 2015.

^116^ S. Schiesser, interview with Fedor Ilich Shishigin on May 18, 2015.

^117^ S. Schiesser, field diary, April 2015.

^118^ Bagheri, “What Qualitative GIS Maps Tell”.

^119^ Granberg and Peresypkin, “*Problemy severnogo morskogo puti”.*

^120^ Laruelle, “Severnyy morskoy put’,” 388.

^121^ Ragner, *Northern Sea Route,* 55, 97.

^122^ Farre et al. “Commercial Arctic Shipping”.

^123^ The project **“** *Russian Harbors of Transarctic Route: Space and Societies of Russia’s Arctic Coast on the Eve of a New Period in the History of Northern Sea Route* ***”*** (2017–2019) is supported by the Russian Ministry of Education and Science; see details in Gavrilova, Vakhtin and Vasil’eva, this issue.

^124^ Salazar, “Polar Infrastructures,” 375.

^125^ Hastrup, “Scales of Attention in Fieldwork”.

^126^
*AHDR*; *AHDR*-*II*.

^127^ Ingold, *The Perception of the Environment*.

^128^ Hughes, *Human*-*Built World*.

^129^ See, e.g. Tsing, “More-than-Human Sociality”.

^130^ See, e.g. Latour, *Reassembling the Social*; and Law, “Actor Network Theory and Material Semiotics”.
